# Reduced-Cost Second-Order Algebraic-Diagrammatic Construction Method for Core Excitations

**DOI:** 10.1021/acs.jctc.3c00101

**Published:** 2023-05-03

**Authors:** Dávid Mester, Mihály Kállay

**Affiliations:** † Department of Physical Chemistry and Materials Science, Faculty of Chemical Technology and Biotechnology, Budapest University of Technology and Economics, Műegyetem rkp. 3, H-1111 Budapest, Hungary; ‡ ELKH-BME Quantum Chemistry Research Group, Műegyetem rkp. 3, H-1111 Budapest, Hungary; § MTA-BME Lendület Quantum Chemistry Research Group, Műegyetem rkp. 3, H-1111 Budapest, Hungary

## Abstract

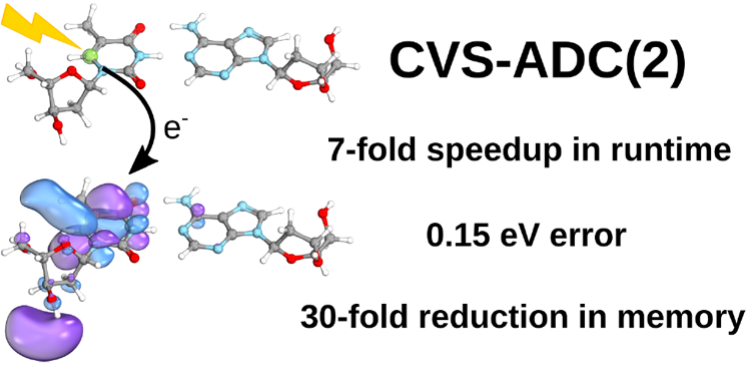

Our reduced-cost scheme [*J. Chem. Phys.***2018**, *148*, 094111] based on the frozen virtual natural orbital and natural auxiliary function approaches is extended to core excitations. The efficiency of the approximation is presented for the second-order algebraic-diagrammatic construction [ADC(2)] method invoking the core–valence separation (CVS) and density fitting approaches. The errors introduced by the present scheme are comprehensively analyzed for more than 200 excitation energies and 80 oscillator strengths, including C, N, and O K-edge excitations, as well as 1*s* → π* and Rydberg transitions. Our results show that significant savings can be gained in computational requirements at the expense of a moderate error. That is, the mean absolute error for the excitation energies, being lower than 0.20 eV, is an order of magnitude smaller than the intrinsic error of CVS-ADC(2), while the mean relative error for the oscillator strengths is between 0.06 and 0.08, which is still acceptable. As significant differences for different types of excitations cannot be observed, the robustness of the approximation is also demonstrated. The improvements in the computational requirements are measured for extended molecules. In this case, an overall 7-fold speedup is obtained in the wall-clock times, while dramatic reductions in the memory requirements are also achieved. In addition, it is also proved that the new approach enables us to perform CVS-ADC(2) calculations within reasonable runtime for systems of 100 atoms using reliable basis sets.

## 1 Introduction

Modern experimental devices, such as synchrotrons and radiation sources of free-electron lasers,^1,2^ allow us to explore the high-energy X-ray region with sufficient accuracy. Accordingly, X-ray spectroscopy is one of the most emerging element-specific tools to obtain chemical information. These characterization techniques are widely used in many fields of science, for example, biochemistry, organic electronics, and nanotechnology.^3−6^

The growing popularity of experimental instruments inspires the development of state-of-the-art theoretical approaches as well, which are essential to understanding and supporting complex measurements. Since extended molecular systems are often studied in the aforementioned fields, the available quantum chemical tools for such demanding simulations are currently limited. In general, time-dependent density functional theory (TDDFT)^7−9^ is the method of choice for excited-state calculations since its computational requirements are relatively low, while the results obtained are in line with experimental measurements in most cases. The formalism was extended to core excitations by Stener and co-workers^10^ invoking the so-called core–valence separation (CVS) approximation.^11^ Using the resulting framework, such methods can be routinely applied to molecular systems of greater than 150 atoms; however, it is well-known that the self-interaction error and the lack of orbital relaxation lead to a strong underestimation of the energies of core-excited states.^12−15^ In addition, the quality of other excited-state properties, such as oscillator strengths, is also frequently in question. To improve the CVS-TDDFT results, several promising approaches have been elaborated in recent years.^16−23^

In general, more consistent and accurate results can be expected from the correlated wave function-based approaches.^24−30^ Among the theories, the algebraic-diagrammatic construction (ADC)^31^ and coupled-cluster (CC)^32^ schemes are the most popular ones. To tackle core excitations, the CVS approximation was first combined with the ADC formalism,^11,33^ while it was later extended to the CC scheme as well.^34^ In the past few decades, several well-established and popular methods have been developed within these theories, such as the second-order CVS-ADC [CVS-ADC(2)] pioneered by Dreuw and co-workers.^35−41^ The reliable performance of this fifth-order scaling method was demonstrated for core excitations in excellent studies,^15,37,39,42^ and a good agreement between the calculations and experiments was revealed. Another notable approach within the theory is the extended variant of CVS-ADC(2) [CVS-ADC(2)-x].^43^ The systematically improvable CVS-ADC formalism enables the indirect inclusion of orbital relaxation effects via couplings to higher-excited configurations. In this case, the doubles–doubles block of the secular matrix is treated up to the first order, while its elements are expanded only in the zeroth order for the strict variant.^15,39,44^ The hierarchical CC scheme also enables the consideration of excitations with arbitrary accuracy. One of the most popular of such methods is the frozen-core equation-of-motion CC singles and doubles (fc-CVS-EOM-CCSD) approach proposed by Coriani et al.,^45^ while other higher-order CC approaches were also developed for highly accurate calculations.^46,47^

As was mentioned, arbitrary accuracy can be attained using the previous schemes; nevertheless, the computational requirements of the high-accuracy methods impose serious limitations in practice. The corresponding sixth-order scaling methods, such as CVS-ADC(2)-x and fc-CVS-EOM-CCSD, are considered as standard approaches for medium-sized molecules of up to 20 atoms, while the CVS-ADC(2) method is recommended for more extended systems of up to 60 atoms. To make CVS-ADC(2) competitive with the less robust CVS-TDDFT methods for large molecules, effective approximations are needed to reduce computation times and storage requirements. For this purpose, several approximations have been developed for valence-excited states; however, their application for core excitations is very limited. In the following, some of these well-established approaches are discussed.

One of the most common bottlenecks is related to the processing of four-center electron repulsion integrals (ERIs), which can be eliminated by invoking density fitting (DF)^48−51^ or Cholesky-decomposition techniques.^52,53^ In these cases, a more compact representation of the ERIs is feasible, which greatly facilitates the storage requirements. Two other related methods are the tensor hypercontraction^54−56^ and the natural auxiliary function (NAF)^57−59^ schemes, where an even lower-order representation of the ERI tensor is used. Another simple technique is the restricted virtual space (RVS) approach,^60,61^ where the high-lying virtual molecular orbitals (MOs) are neglected. None of these approximations use any information about the excitation. Significantly more efficient schemes can be developed by determining the MOs that play an important role in the transitions. In this class, the most popular approaches are primarily based on the frozen natural orbital (NO) approximation.^58,59,62−68^ Here, a one-particle density matrix is constructed using a more approximate method, such as the configuration interaction singles (CIS)^69^ approach with perturbative second-order correction [CIS(D)].^70^ Thereafter, the matrix is diagonalized, and the resulting NOs with small occupation numbers are neglected. Further improvements in dimension reduction can be achieved if the locality of the MOs is also exploited.^71^ The first local excited-state approach was presented by Crawford et al.,^72−74^ while several advances were reported later by different groups. In this direction, one of the most significant results was achieved by Korona, Schütz, and their co-workers.^75−77^ Their efficient Laplace transform-based multistate approach was also extended to analytic energy gradient calculations.^78,79^ Parallel to those efforts, promising schemes were also proposed by Hättig et al. extending the pair natural orbital (PNO) approximation to excited-state theories,^80−82^ while further PNO-based approaches were presented by Dutta and co-workers^83^ and Crawford et al.^84^ We note that other approximations are also available to reduce the computational requirements, such as the multilevel schemes developed by Koch and co-workers.^85,86^

The usage of the aforementioned approximations for core excitations is rather limited. First, multilevel-based approaches were proposed by Koch and co-workers.^87−89^ Thereafter, the RVS scheme was extended to such calculations by Dreuw et al.,^41^ while the frozen virtual NO (VNO) approximation was tested by Krylov and co-workers.^90^ Recently, a PNO-based CVS-EOM-CCSD method was proposed by Dutta et al.^91^ for core binding energies. We note that cost reduction techniques were also reported for CVS-TDDFT calculations, such as the auxiliary density matrix method approximation.^92^

In this paper, our reduced-cost ADC(2) scheme^59^ based on the VNO and NAF approximations is extended to core excitations. Using the recently proposed XABOOM benchmark set,^42^ a representative test set including more than 200 excitation energies and 80 oscillator strengths is compiled. The errors introduced by the present scheme are comprehensively analyzed for this compilation, while the efficiency is also demonstrated for extended molecular systems.

## 2 Theory

### 2.1 CVS-ADC(2) Approach

For core excitations, one of the best trade-offs between accuracy and computational requirements is offered by the CVS-ADC(2) approach.^11,37,41^ As this well-established method and its theoretical background have been comprehensively discussed in excellent studies,^37−41^ only a brief overview of the working equations and algorithmic considerations is presented herein. The expressions for CVS-ADC(2) are very similar to those that appears in ADC(2); therefore, their implementation is based on our existing ADC(2) framework^59^ inspired by the original work of Hättig and Weigend.^51,93^ In this approach, a nonlinear eigenvalue equation,
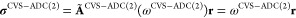
1is solved, where  is the so-called effective Jacobian, **r** denotes the singles excitation vector, and ω^CVS–ADC(2)^ is the ADC(2) excitation energy within the CVS approximation. The benefit of the above pseudo-eigenvalue equation is that the resulting problem has to be only solved in the space spanned by the single excitations, while the doubles amplitudes can be calculated on the fly, and their storage can be avoided. An important feature of the effective Jacobian is that it depends on the excitation energy of the given excited state. Thus, using the conventional Davidson-type diagonalization techniques,^94,95^ the eigenvalue equation cannot be solved simultaneously for all the considered states. To remedy this issue, a procedure using a modified Davidson algorithm and the direct inversion in the iterative subspace^96^ acceleration was suggested in ref (51). The most time-consuming step of the procedure is the sigma vector construction defined by eq 1. The vector can be split into two parts as
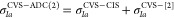
2where  is the CVS-CIS sigma vector, and all the expressions including second-order contributions are merged into . The elements of  are obtained as
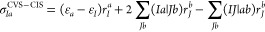
3where ε_*p*_ stands for a corresponding MO energy and (*Ia*|*Jb*) is a four-center ERI using the Mulliken convention. The notation follows the convention that *I*, *J*, ... and *i*, *j*, ... are the active (core) and inactive occupied molecular orbital indices, respectively, whereas *a*, *b*, ... (*p*, *q*, ...) denote virtual (generic) orbitals. Furthermore, the expressions for  read explicitly as
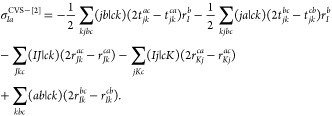
4

The first two terms on the right-hand side contain the contributions of the corresponding ground-state second-oder Møller–Plesset (MP2) amplitudes given by
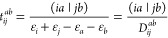
5while the remaining terms are associated with the double excitation coefficients, which can be expressed as
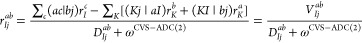
6

Once the corresponding sigma vectors are calculated, the excitation energies are simply obtained by solving eq 1 using standard iterative diagonalization techniques, such as the Davidson or Lanczos algorithm. To calculate oscillator strengths, a linear-response CC consistent transition density matrix^97,98^ is employed. The explicit expressions using the CVS approximation have been presented in ref (99).

Introducing the DF approach, a computationally favorable algorithm can be designed, where the ERIs are approximated as the products of two- and three-center arrays:
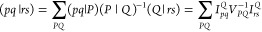
7where *P* and *Q* stand for the elements of the auxiliary basis, while  and *V*_*PQ*_ are three- and two-center Coulomb integrals, respectively, and  is a simplified notation for the corresponding element of the inverse of the two-center Coulomb integral matrix. Usually, this inverse matrix is factorized and rewritten; therefore, the ERIs can be recast as **IV**^–1/2^**V**^–1/2^**I**^T^ = **JJ**^T^, where the final form of the three-center integrals reads explicitly as
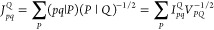
8

Utilizing the expressions obtained, an efficient algorithm can be designed to calculate eqs 3 and 4. To the best of our knowledge, such working equations have not been published in the literature so far. Accordingly, the steps of the procedure are given in detail in Table 1. Inspecting the scaling of the various operations, we can conclude that the rate-determining steps in the iterative part are the construction of the double excitation coefficients and their contraction with the corresponding three-center integrals (step 3). The operation count for these steps is proportional to , where *n* denotes the number of the corresponding orbitals and functions. As *n*_*I*_ and *n*_*i*_ are usually small, the demands are mainly affected by *n*_*a*_ and *n*_*Q*_. Thus, if an efficient approximation could be developed to shrink these dimensions, then significant savings in computational costs can be achieved. In our previous work, inspecting valence-excited states, the frozen VNO and NAF approximations have been suggested for this purpose.^59^

**Table 1:** Algorithm and Working Equations for Calculating the Sigma Vector

0. Perform a frozen-core MP2 calculation and store intermediate *X*_*ab*_
, where
1. Calculate intermediates *W*^*Q*^ and

2. Add CVS-CIS contributions to σ_*Ia*_

3. Construct and build intermediates and




4. Add double excitation contributions to σ_*Ia*_

5. Add ground-state amplitude contributions to σ_*Ia*_


### 2.2 Frozen VNO and NAF Approximations

To decrease the dimension of the MO space, one of the most well-established approaches is the frozen NO approximation.^62−64^ On the basis of our previous experiences for valence-excited states, significant reductions can be achieved for virtual orbitals, while the benefits are more moderate for the occupied orbitals.^58^ Therefore, the present discussion is restricted to the application of frozen VNOs. In this case, the virtual–virtual block of the one-particle density matrix,
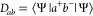
9is constructed, where Ψ denotes a corresponding lower-level wave function, and *a*^+^ and *b*^–^ are creation and annihilation operators, respectively, acting on the corresponding virtual orbitals. Thereafter, the matrix is diagonalized, and its eigenvectors are referred to as the VNOs, whereas the corresponding eigenvalues are interpreted as the relative importance of the orbitals. It is supposed that the orbitals with lower importance give a smaller contribution to the excitation energy and can be dropped without any significant loss in accuracy. Consequently, using a predefined truncation threshold, let us denote it by ε_VNO_, the most important orbitals are selected, and the remaining VNOs can be safely disregarded. It is essential to keep in mind that the reduced subspace should contain all the VNOs required for the adequate description of the ground and excited states simultaneously. On top of this, it is highly desirable to solve both the ground- and excited-state equations in the same subspace. Accordingly, the proper selection of Ψ is not unequivocal. In our previous studies,^58,59^ it has been demonstrated that state-specific VNOs derived from a so-called state-averaged density matrix are highly suitable for valence-excited states. This density matrix was formed for each transition as **D** = (**D**^MP2^ + **D**^CIS(D)^)/2, where **D**^MP2^ and **D**^CIS(D)^ are the approximate density matrices obtained from the MP2 and CIS(D) wave functions, respectively. The algorithmic considerations to calculate the corresponding densities were discussed in detail in ref (58). It can be supposed that a similar density would be ideal for core excitations as well; however, to simplify the working equations, the CVS approximation can be exploited. Accordingly, **D**^CIS(D)^ is replaced by **D**^CVS–CIS(D)^ in this study. We note that, as state-specific VNOs are used in the present scheme, the strict orthogonality of the excited states is not ensured. In this study, only ground- to excited-state transition moments are concerned, where the nonorthogonality of the excited states does not cause any problem. In addition, the calculation of transition moments between two core-excited states are somewhat less relevant, but their implementation in our framework would require special considerations.

As was discussed in the previous subsection, the number of fitting functions can also be troublesome for extended systems. Utilizing the NAF approximation,^57^ the size of the auxiliary basis set can be effectively reduced via the singular value decomposition of the three-center integral matrix. The approach is very similar to the previously discussed NO approximation; however, it is not based on any physical or chemical consideration. It can be shown that the truncated singular vector basis of **J** is the best approximate auxilary basis for the tree-center Coulomb integrals and hence to the corresponding four-center ERIs in the least-squares sense. In practice, it is more favorable to compute the NAFs as the eigenvectors of matrix **W** obtained as
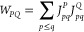
10while the eigenvalues of the matrix are the squares of the singular values and can be interpreted as the importance of the auxiliary functions. Similar to the NO approach, using a predefined threshold denoted by ε_NAF_, the less important NAFs can be dropped to get a more compact representation of **J**.

To conclude this section, all the main steps required for the subspace construction using the combined VNO + NAF scheme are collected in Table 2. We note that the VNO and NAF approximations can be applied separately as well. In the former case, steps 2 and 4.b are omitted, while steps 4.a and 4.b are skipped for the latter one. As can be seen, the NAF basis is truncated twice in the combined scheme. In these steps, the same cutoff parameter is applied. Accordingly, if only the NAF approximation is used, the number of retained NAFs is not changed in step 4.b. For the interested readers, we recommend our previous work,^59^ where the detailed algorithms have been presented for valence-excited states.

**Table 2:** Algorithm for the reduced-cost CVS-ADC(2) approach using the combined VNO + NAF scheme

0. Solve Hartree–Fock equations
1. Construct the canonical three-center integral list
2. Calculate matrix **W** and construct the truncated auxiliary basis
3. Solve CVS-CIS equations for all the excited states simultaneously
4. Loop over excited states
4.a. Calculate the state-averaged one-particle density matrix **D** using three-index integrals obtained in step 2 and construct the truncated virtual space
4.b. Calculate matrix **W** using three-index integrals obtained in the previous step and construct the final truncated auxiliary basis
4.c. Calculate MP2 and solve CVS-ADC(2) equations in the reduced subspace End loop

## 3 Computational Details

### 3.1 Calculation of the Numerical Results

The new reduced-cost CVS-ADC(2) approach has been implemented in the Mrcc suite of quantum chemical programs and will be available in the next release of the package.^100^ The proper basis set selection for core excitations is still an actively studied field.^42,90,101−104^ As was suggested in ref (42), triple-ζ quality basis sets are a highly appropriate choice for 1*s* → π* excitations. However, more diffuse functions may also be required for Rydberg excitations. Accordingly, Dunning’s diffuse function augmented triple-ζ basis sets (aug-cc-pVTZ)^105−107^ were used for all atoms. The DF approximation was utilized in both the ground- and excited-state calculations, and the corresponding auxiliary basis sets of Weigend and co-workers^108−110^ were employed for this purpose. The conventional CVS spaces were used for all of the molecules. The oscillator strengths, denoted by *f*, were computed in the dipole length approximation, and a linear-response CC consistent formalism^97,98,111^ was used for the transition densities invoking the CVS approximation. For the NAF and VNO selection, the default ε_NAF_ = 0.1 au and ε_VNO_ = 7.5 × 10^–5^ cutoff thresholds were used, respectively. As was shown in ref (59), these conservatively selected parameters guarantee that the errors are much smaller than the intrinsic error of the ADC(2) method, at least for valence-excited states. The reported computation times are wall-clock times determined on a machine with 128 GB of main memory and an 8-core 1.7 GHz Intel Xeon E5–2609 v4 processor.

Our main intention in this study is to quantify the errors originating from our approximations. Consequently, we do not endeavor to highlight the strengths and weaknesses of the canonical CVS-ADC(2) method in any respect. Only the intrinsic error of CVS-ADC(2) will be used to measure the accuracy of the approximations. Thus, the computed numerical values are always compared to the canonical ones. The main statistical error measures for the excitation energies are the mean absolute error (MAE) and the maximum absolute error (MAX). For the oscillator strengths, the relative error of the intensities is in focus. In this case, the mean relative error (MRE) and the maximum relative error are discussed in detail. For convenience, the latter measure will also be denoted by MAX. All the computed excitation energies, oscillator strengths, and statistical error measures are available in the Supporting Information (SI). In addition, further measures, such as the mean error, root-mean-square error, standard deviation (SD), and deviation span are also included. In some cases, the SDs will be briefly discussed as a proper measure to quantify the precision of the approximations.

### 3.2 Molecular Systems: The XABOOM Benchmark Set and Other Extended Molecules

**Figure 1:**
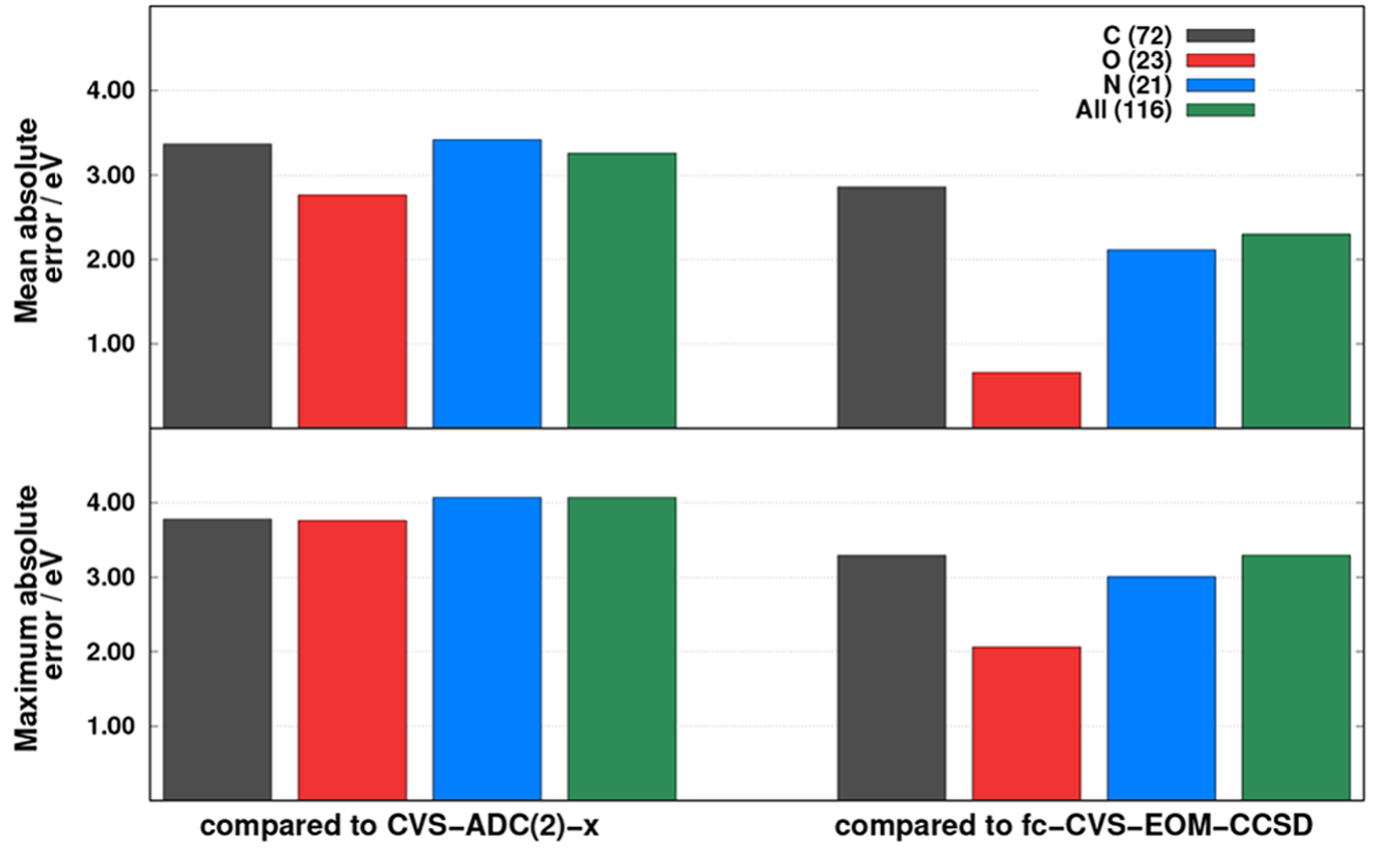
Error measures for the CVS-ADC(2) excitation energies for the C, O, and N K-edge transitions of the XABOOM benchmark set^42^ using the so-called “aT/T/D” basis sets compared to higher-order methods. Values were taken from ref (42). The numbers of transitions are in parentheses.

For the benchmark calculations, molecules from the recently proposed XABOOM^42^ test set were used, which contains 40, primarily organic compounds. The selection of the molecular systems was inspired by the popular benchmark set of Thiel and co-workers;^112^ therefore, it contains unsaturated aliphatic hydrocarbons, heterocycles, aromatic hydrocarbons, carbonyl compounds, nucleobases, etc. Unfortunately, the test set only incorporates 1*s* → π* transitions with sharp absorption bands. To quantify the intrinsic error of CVS-ADC(2) for such excitations, error measures for the XABOOM benchmark set are collected in Figure 1. As can be seen, despite the similarity of CVS-ADC(2) and CVS-ADC(2)-x, a somewhat higher accuracy was obtained if fc-CVS-EOM-CCSD values are selected as the references. However, in this case, the errors significantly depend on the excitation type. That is, the MAE is around 3.00 eV for the C K-edge transitions, while it is 2.00 and 0.70 eV for the N and O K-edge excitations, respectively. Similar findings can be obtained if the MAXs are inspected. The most considerable discrepancies are observed for the C and N K-edge excitations, with MAXs of about 3.00 eV, whereas it is 2.00 eV for the O K-edge transitions. The errors are well-balanced if CVS-ADC(2)-x references are considered. In this case, the MAEs are around 3.00 eV, while the MAXs are about 4.00 eV for all types of K-edge excitations. Presumably, as was demonstrated for valence-excited states, a somewhat more notable inaccuracy can be expected for Rydberg transitions.^113^ Our primary purpose is to keep the overall error introduced by the present approximations an order of magnitude smaller than the intrinsic error of CVS-ADC(2). Thus, on the basis of these numerical experiences, an accuracy of 0.20 eV would be highly acceptable.

To select the core-excited states considered in this study, a quite different strategy was used than in ref (42). For all the molecules, the three lowest C, O, and N K-edge excitations were calculated if the corresponding heavy atom is present in the compounds. This choice ensures that an appropriate amount of Rydberg excitations will also be included in the benchmark set. Thereafter, in the case of degenerate states, one of the corresponding states was excluded from the statistics. The resulting compilation contains 124 1*s* → π* and 87 Rydberg excitations, as well as 101 C, 58 O, and 52 N K-edge transitions that were considered.

**Figure 2:**
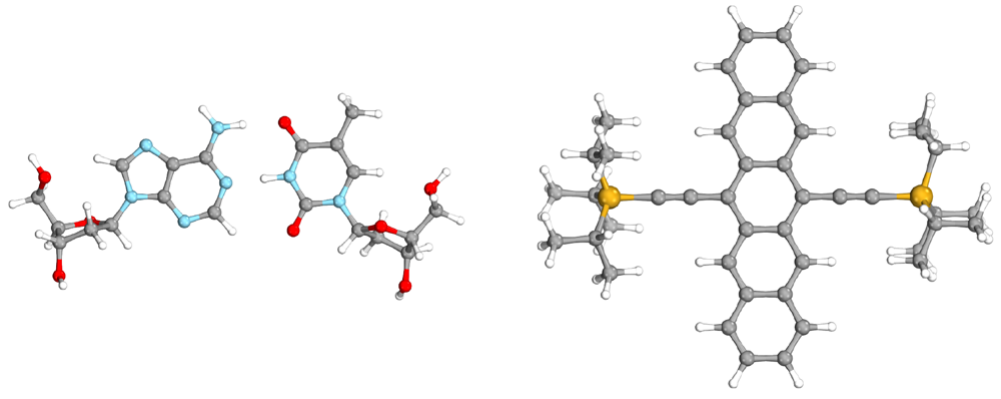
Structures of the extended molecules: DNA_1_ (left) and TIPS-pentacene (right).

To demonstrate the efficiency of our scheme, additional calculations were also carried out for extended molecular systems from the field of biochemistry and organic electronics. For this purpose, a single DNA adenine-thymine base pair (DNA_1_)^114^ and the TIPS-pentacene molecule were selected. The role and importance of these model systems have been summarized in excellent studies.^115−128^ The ground-state geometries of the molecules were optimized at the GFN2-xTB level.^129^ The resulting structures are presented in Figure 2, while the corresponding Cartesian coordinates are available in the SI. The molecules are visualized by the IboView program.^130,131^

## 4 Results

### 4.1 Accuracy of the Approximations

#### 4.1.1 Excitation Energies

**Figure 3:**
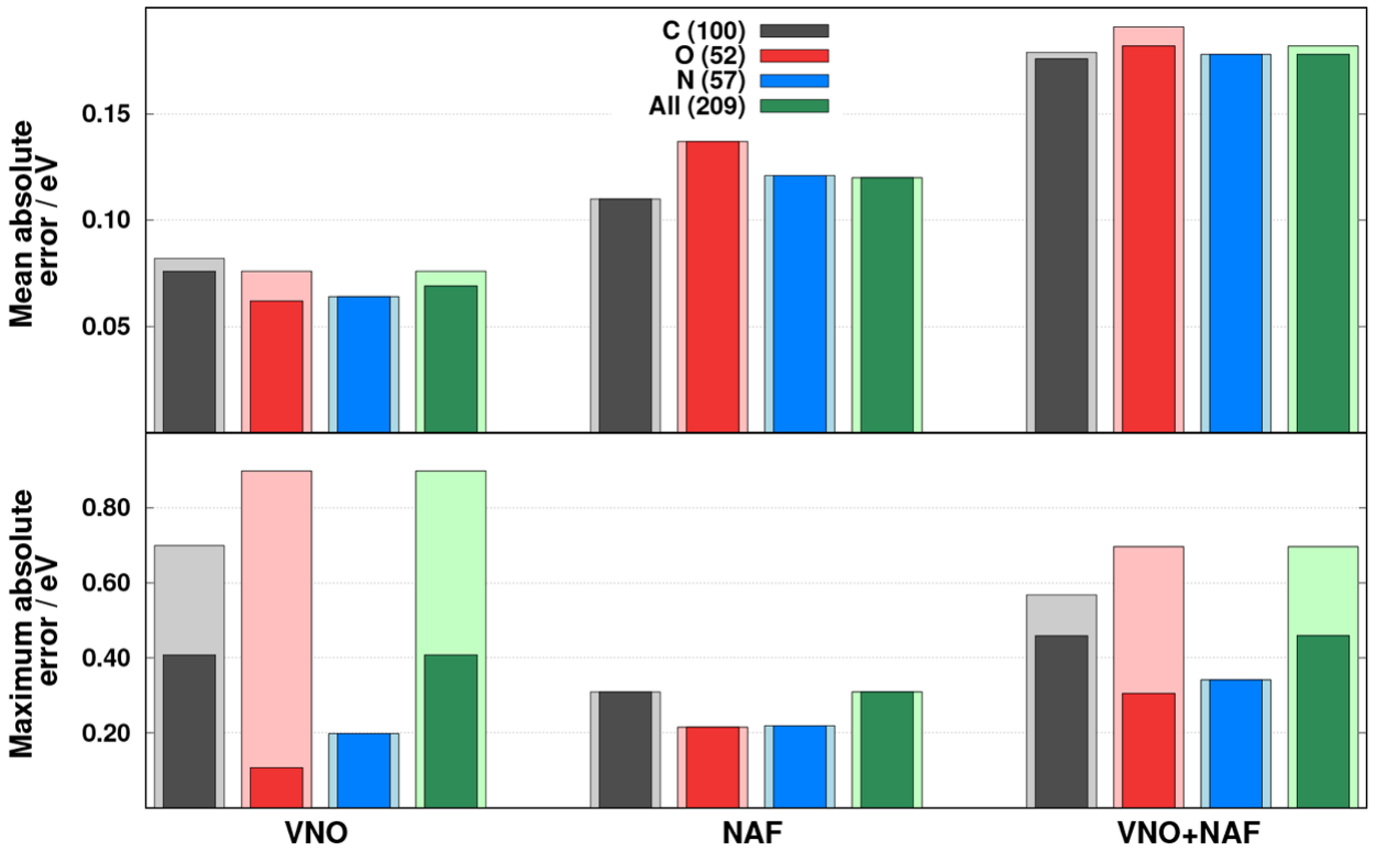
Error measures for the excitation energies using different approximations. The darker bars indicate when the first C K-edge excitation of C_2_FH_3_ and the third O K-edge excitation of N_2_O are excluded. The numbers of transitions are in parentheses.

First, we study the accuracy of our approximations for excitation energies using the default cutoff parameters. The accuracy of VNO and NAF approximations is inspected separately, while their combination, as the most effective approach for dimension reduction, is also assessed. The error measures obtained for the previously discussed benchmark set are presented in Figure 3.

Inspecting the results, two salient errors can be identified if the VNO approximation is invoked: the first C K-edge excitation of C_2_FH_3_ and the third O K-edge excitation of N_2_O. As expected, the VNO selection is only ideal for CVS-ADC(2) calculations if the CVS-CIS and hence the CVS-CIS(D) methods provide a qualitatively good description of the excited state. Furthermore, even the performance of CVS-ADC(2) is questionable. In these particular cases, we found that the overlap of the CVS-CIS and the single excitation part of the final CVS-ADC(2) wave functions is extremely low. Accordingly, a couple of the virtual orbitals with significant contributions to the CVS-ADC(2) wave function are missing in the reduced space. As can be seen, even though the approximation introduces a notable error, only less than 1% of the transitions are affected. If these two states are excluded from the statistics, then the outcomes barely change concerning the overall performances; of course, it has a more significant effect on the maximum errors. Nevertheless, the detailed discussions regarding the performances will ignore these two irregular excitations.

On the basis of the numerical results obtained, we can conclude that the MAEs within a given approximation are well-balanced for different types of excitations. That is, if only the VNO approach is utilized, the MAEs are around 0.06 eV for the O and N K-edge excitations, while it is 0.08 eV for the C K-edge transitions. The overall errors are somewhat less favorable when only the NAF approximation is used. This observation is quite surprising, as the approach proved to be practically error-free in our previous work.^58^ Nevertheless, despite the unexpected results, the MAEs are still acceptable. In this case, the lowest MAE, being around 0.10 eV, is observed for the C K-edge excitations, while the overall errors are 0.12 and 0.14 eV for the N and O K-edge transitions, respectively. As expected, the highest errors are obtained when the VNO and NAF approximations are combined. In this case, practically identical errors were measured for different types of excitations. The MAEs are around 0.18 eV, which is still acceptable in comparison with the intrinsic error of CVS-ADC(2). In addition to accuracy, precision is also worth discussing for the VNO + NAF approximation. In contrast to the MAEs, the SDs are somewhat more sensitive for the element-specific excitations. The lowest deviation, precisely 0.03 eV, is attained for the O K-edge transitions, while it is only 0.05 eV for the N K-edge excitations. Less satisfactory results were obtained for the C K-edge transitions, where the SD is 0.10 eV; however, it is still moderate compared with the original precision of the CVS-ADC(2) method.^42^

Inspecting the MAXs, the highest errors are obtained for the C K-edge excitations. If only the VNO approximation is applied, then the error measure is around 0.40 eV for such transitions, while the MAXs are more moderate for the N and O K-edge excitations, with discrepancies of 0.20 and 0.10 eV, respectively. For the NAF approach, these values are somewhat more balanced. That is, the MAXs are 0.22 eV for the N and O K-edge excitations, while it is 0.31 eV for the C K-edge transitions. As can be seen, a practically identical value is measured for the N K-edge excitations, while the MAX is slightly more (less) favorable for the O (C) K-edge transitions compared to the frozen VNO results. Again, the highest values are obtained for the VNO + NAF approximation; however, the differences are not so significant. The MAX is around 0.45 eV for the C K-edge excitations, while they are below 0.35 eV for the N and O K-edge transitions.

#### 4.1.2 Robustness for Different Types of Excitations

**Figure 4:**
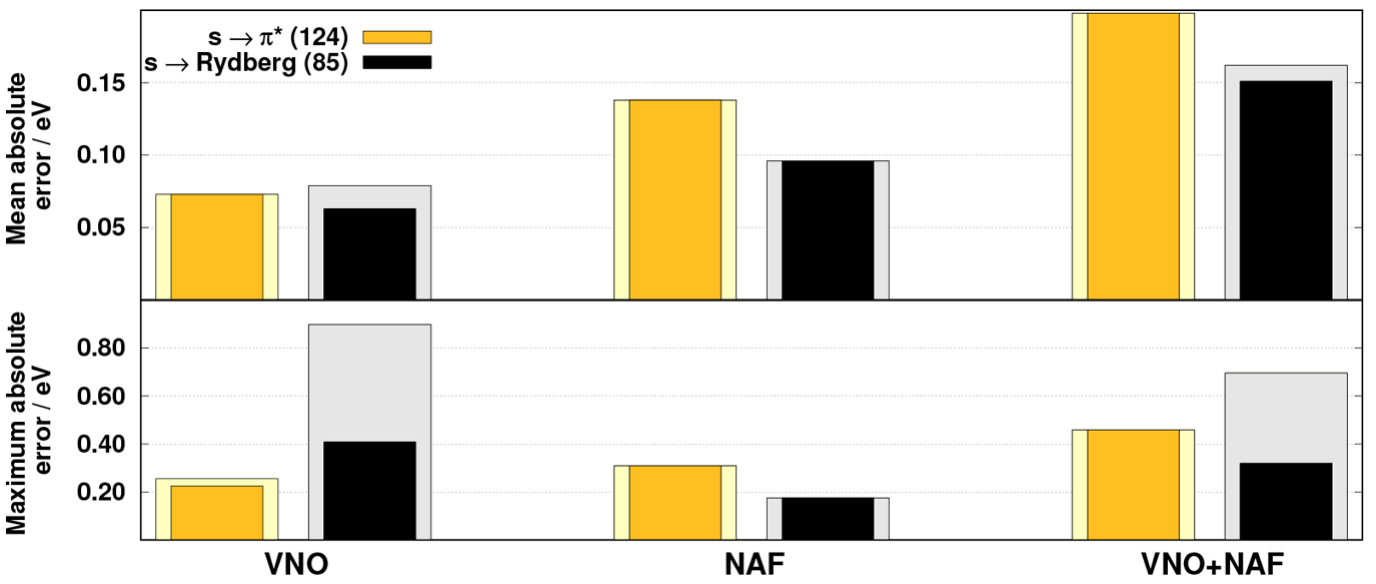
MAEs for the *s* → π* and *s* → Rydberg transitions. The darker bars indicate when the first C K-edge excitation of C_2_FH_3_ and the third O K-edge excitation of N_2_O are excluded. The numbers of transitions are in parentheses.

Next, the accuracy for different characters of transitions is assessed separately. The results obtained for the 1*s* → π* and Rydberg excitations are collected in Figure 4. As can be seen, a similar accuracy is expected if only the VNO approximation is applied. Thus, the overall errors are practically identical in this case, with MAEs of around 0.07 eV. The difference is somewhat more notable when only the NAF approach is used. That is, the MAE is still below 0.10 eV for the Rydberg transitions, while it is 0.14 eV for the 1*s* → π* excitations. This trend persists for the combined VNO + NAF approximation as well. The overall error is 0.15 eV for the Rydberg excitations, while the MAE is 0.19 eV for the latter ones. Interestingly, the opposite tendency can be observed if the SDs are inspected. In this case, the deviation is 0.07 eV for the 1*s* → π* transitions, while a less favorable SD, precisely 0.09 eV, is attained for the Rydberg excitations. On the basis of these results, we can conclude that the combined VNO + NAF approximation is a bit more accurate if Rydberg orbitals are involved in the excitation, while slightly more precise results are expected for 1*s* → π* transitions. However, the differences, being around 0.04 and 0.02 eV, respectively, are not significant. If only the VNO approach is applied, the largest error, precisely 0.40 eV, is affiliated with a Rydberg excitation, while it is significantly milder for the 1*s* → π* transitions. The MAXs are more balanced for the NAF approximation. In this case, the largest errors are 0.31 and 0.18 eV for the 1*s* → π* and Rydberg excitations, respectively. Concerning the combined VNO + NAF approach, the largest error belongs to a 1*s* → π* transition, with a MAX of 0.46 eV, while it is 0.32 eV for the Rydberg excitations.

#### 4.1.3 Oscillator Strengths

**Figure 5:**
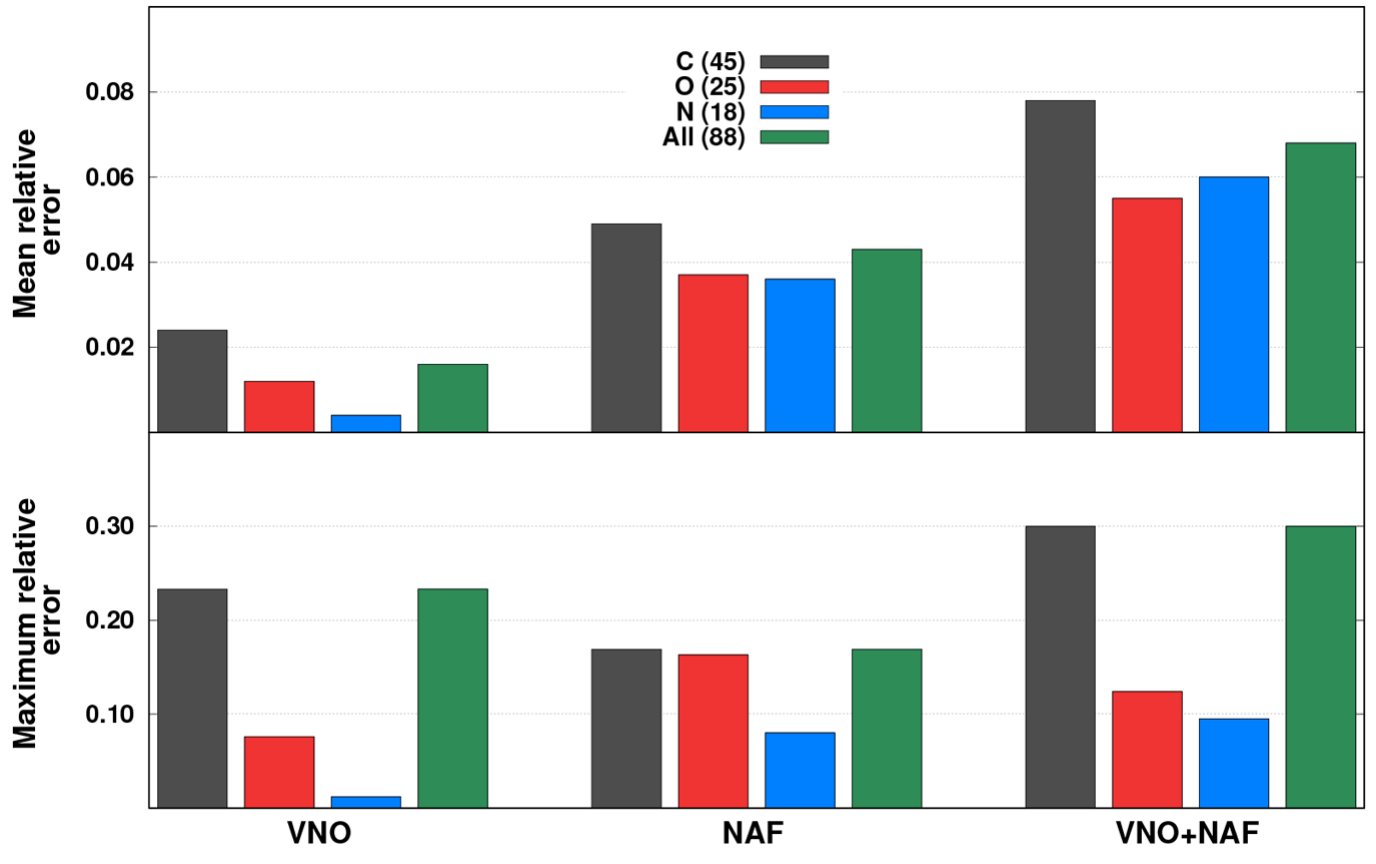
Error measures for the oscillator strengths (*f* > 0.015) using different approximations. The numbers of transitions are in parentheses.

Finally, the oscillator strengths are discussed in detail. The error measures, where transitions only with *f* > 0.015 are included in the statistics, are presented in Figure 5. Inspecting the error bars, we can conclude that the MREs are decent if only the VNO approach is used. The calculated oscillator strengths are almost error-free for the N K-edge excitations, while the MRE is only 0.012 for the O K-edge transitions. A significantly larger value, being around 0.023, is obtained for the C K-edge excitations; however, the magnitude of the error is still negligible. The results are more balanced if the NAF approximation is applied. The MREs are about 0.036 for the N and O K-edge excitations, while it is 0.050 for the C K-edge transitions. As can be seen, again, the errors are more notable in these cases in comparison with the frozen VNO results. It is also true if the combined VNO + NAF approximation is used. The MREs are about 0.060 for the N and O K-edge excitations, while it is just below 0.080 for the C K-edge transitions. Considering the results obtained for the original XABOOM test set,^42^ where MREs of around 0.120 and 0.220 were measured against fc-CVS-EOM-CCSD and CVS-ADC(2)-x references, respectively, we can conclude that the oscillator strengths are somewhat more affected by the approximations than the excitation energies. This is in line with the results obtained for valence-excited states,^59^ while a similar trend was also revealed for frequency-dependent dipole polarizabilities by Kumar and Crawford.^66^ Inspecting the MAXs, one salient error can be identified if only the VNO approximation is applied. In this case, the MAX is affiliated with a C K-edge excitation, while the discrepancies are considerably milder for the remaining types of excitations. If only the NAF approach is utilized, then the MAX does not exceed 0.080 for the N K-edge transitions, while they are just below 0.170 for the C and O K-edge excitations. If the combined VNO + NAF approach is used, highly acceptable results are reached for the N and O K-edge excitations, with MAXs of 0.095 and 0.124, respectively. However, a significantly higher error, precisely 0.300, is obtained for the C K-edge transitions. Concerning the canonical ADC(2) results, we note that MAXs around 0.500 and 0.600 were obtained for the XABOOM test set using fc-CVS-EOM-CCSD and CVS-ADC(2)-x references, respectively.^42^

### 4.2 Compression of the Dimensions

#### 4.2.1 General Considerations

**Figure 6:**
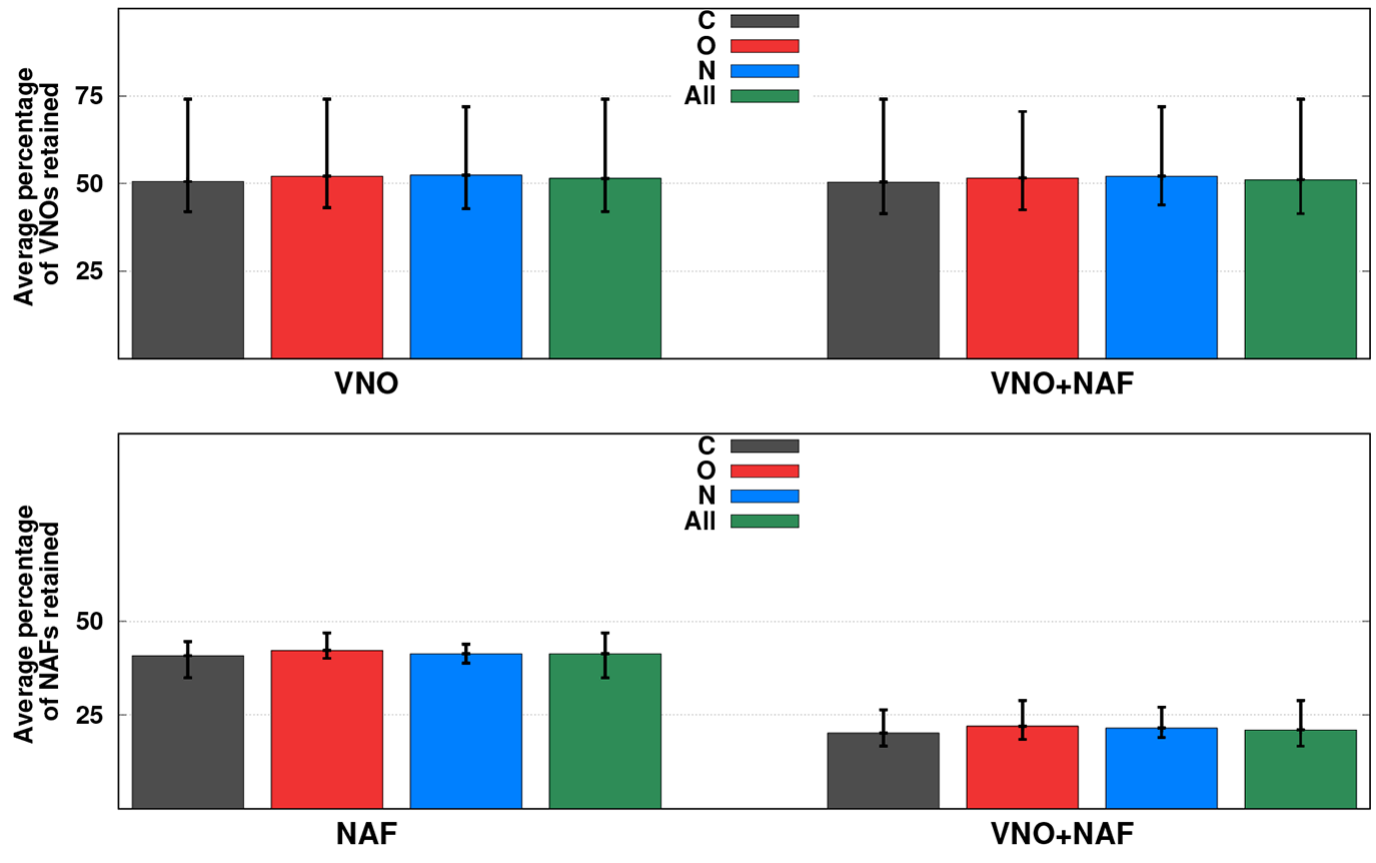
Percentage of VNOs and NAFs retained. The smallest and largest values are indicated on the bars.

The above results show the accuracy and precision of the approximations, but the computational benefits are also important. To shed some light on this aspect, the corresponding basis set reductions regarding the VNOs and NAFs are collected in Figure 6. As can be seen, practically the same statistical measures are obtained for all the K-edge transitions regardless of the elements. Therefore, only the overall results are discussed. First, the VNO approach is inspected in detail. In this case, on average, almost 50% of the VNOs can be safely neglected, while the maximum and minimum subspace reductions are 58 and 26%, respectively. On the basis of our previous experiences,^58,59^ this notable difference between the minimum and maximum values is somewhat surprising. However, as we will see, the lowest values belong to di- and triatomic systems, while the truncations are reasonably constant for larger systems. Concerning the NAF approximation, only 41% of the auxiliary functions are retained in this case, and the reductions fluctuate between 53 and 65%. Thus, the difference between the extrema is significantly lower, and the gains are more balanced than those for the frozen VNO approach. The most effective approximation for dimension compression is the combined VNO + NAF approach. We would like to point out that the NAF approximation affects the accuracy of all quantities that are computed subsequently (see in Table 2), such as one-particle density matrices, VNOs, etc. Accordingly, the number of VNOs in the reduced subspace may differ compared with the genuine VNO approach. However, as can be seen, the discrepancy is negligible. That is, the overall subspace truncation regarding the VNOs is larger by 0.4% in this case, while the extrema are also affected to this extent. Alternatively, the NAF basis is significantly more compressed than that for the standalone NAF approximation. As expected, in the truncated VNO basis, considerably fewer auxiliary functions are necessary for similar accuracy. In this case, on average, almost 80% of the NAFs are neglected, while the maximum and minimum truncations are 71 and 83%, respectively. The benefits of the combined VNO + NAF approach over the VNO approximation are twofold. First, as about 60% of the auxiliary functions are dropped at the beginning of the procedure, most of the rate-determining steps required for the VNO construction can be performed about 60% faster. Second, as can be seen, the final NAF basis can be extremely compressed in the truncated VNO subspace. On the basis of the numerical results, these reductions yield a 20-fold cut in the operation count for the ADC(2) part, while the memory requirement can also be reduced to the same extent.

#### 4.2.2 Robustness with Increasing System Size

**Figure 7:**
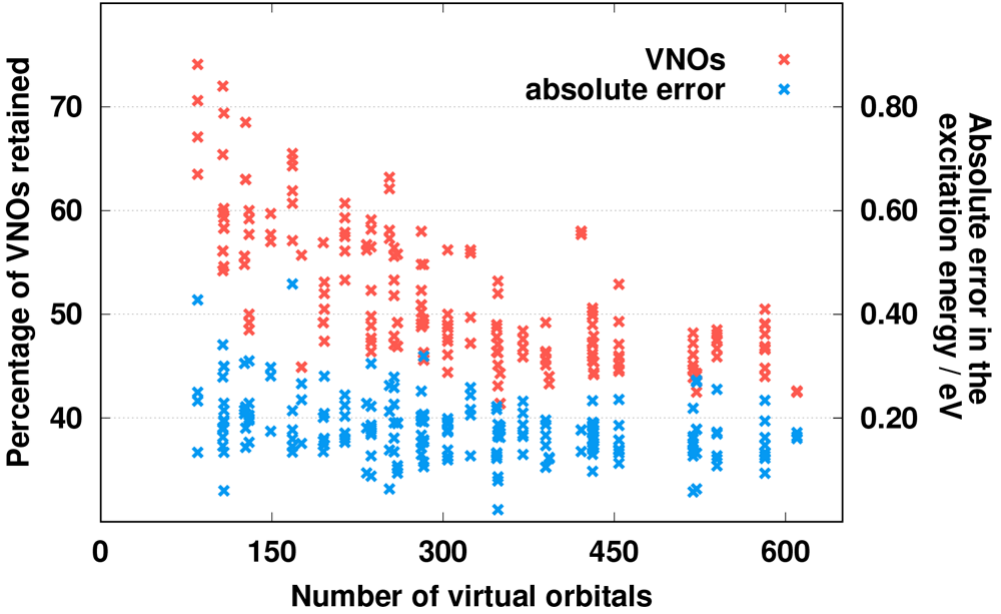
Percentage of VNOs retained (red) and the absolute error in the excitation energy (blue) as a function of the system size.

Next, the dependence of the results is analyzed regarding the size of the molecular system. It is trivial that the number of canonical virtual orbitals and auxiliary functions increases when going toward the larger systems. Hence, the number of dropped VNOs and NAFs increases as well. Accordingly, it is important to point out that the errors introduced by the approximations do not grow with increasing system size. On top of this, as was mentioned, the difference between the minimum and maximum VNO truncations for the benchmark calculations may seem notable. In this short study, we would also like to prove that the percentage of the retained VNOs fluctuates within a narrow range from a given point. To this end, the obtained results for each excitation in the benchmark compilation, using the combined VNO + NAF approach, are plotted in Figure 7. Inspecting the trends, we can conclude that the errors barely change with the system size; perhaps the results are somewhat more favorable for larger molecules. That is, the errors become slightly more balanced with increasing system size, and their averages are also somewhat more moderate for extended compounds. Similar findings can be observed for the proportion of the retained VNOs. In this case, the truncations fluctuate within a wide range for small molecules. The difference between the lowest and highest values can be almost 25%. This range decreases rapidly with increasing system size, and the difference is less than 10% for extended systems including more than 500 virtual orbitals. In other words, similar and well-predictable truncations can be achieved for larger compounds, while the expected errors are unaffected by system size or even more favorable for extended molecules.

### 4.3 Extended Molecules

#### 4.3.1 Speedups

Our approach was further tested for extended molecules to demonstrate its efficiency. So far, for this purpose, we have reported the percentage of the retained VNOs and NAFs and determined the expected operation count reduction at certain steps of the algorithm. An even better measure is to compare the factual wall-clock times obtained for the conventional and the reduced-cost CVS-ADC(2) approaches. To this end, benchmark calculations were carried out for the four lowest C, N, and O K-edge excitations of DNA_1_. This medium-sized system contains 62 atoms and 2231 atomic orbitals using the aug-cc-pVTZ basis set. The numerical results are collected in Table 3. First, the conclusions drawn for the smaller systems are briefly checked. As can be seen, a moderate overall error is achieved for the excitation energies, with a MAE of 0.14 eV, while the MRE is 0.08 for the oscillator strengths. Notable differences between the errors obtained for the C, N, and O K-edge excitations cannot be observed. These findings are highly in line with the results presented for the benchmark set. In addition, on average, 42% of the VNOs were retained, while the NAF basis was compressed by a factor of 5. These reductions are a bit larger than those for smaller molecules; however, as was demonstrated, the truncations slightly increase with increasing system size. Inspecting the speedups, we can conclude that an overall speedup factor of 7 can be obtained for such systems. Some correlation between the improvements and the number of occupied orbitals can be observed. That is, on the basis of the truncations, the relative operation count reductions in the ADC(2) part are similar for all types of excitations. However, the factual improvements are more favorable when the ADC(2) part is more expensive as only this step is treated in the reduced subspace. Accordingly, as the *n*_*I*_ × *n*_*i*_ value is significantly larger for the C K-edge excitations, the overall speedup is more notable in this case, while the improvements are slightly smaller and more balanced for the remaining types of core excitations. In these cases, the *n*_*I*_ × *n*_*i*_ values are practically identical, and the canonical ADC(2) part is relatively less demanding. Nevertheless, the differences are reasonably small, and the speedups are well-balanced. In addition, considering the wall-clock times measured, one can see that the calculation takes less than half a day for the four lowest core excitations. This demonstrates that such calculations can be routinely carried out for extended molecular systems with more than 60 atoms.

**Table 3:** Canonical CVS-ADC(2) Excitation Energies (ω, in eV) and Oscillator Strengths (*f*) for DNA_1_ Using the aug-cc-pVTZ Basis Set; As Well As the Corresponding Error Measures, Percentage of VNOs and NAFs Retained, Total Wall-Clock Times (in Hours), and Overall Speedups with the Reduced-Cost Algorithm^a^

K-edge	state	character	ω	*f*	abs. error in ω	rel. error in *f*	retained VNOs	retained NAFs	total wall time	speedup
C	1	*s* → π*	288.11	0.0270	0.13	0.07	41.0	17.9	11.4	7.7
	2	*s* → π*	289.25	0.0593	0.20	0.06	41.0	17.9		
	3	Rydberg	289.39	0.0126	0.10		42.3	18.0		
	4	Rydberg	289.60	0.0138	0.10		42.3	18.0		
N	1	Rydberg	401.98	0.0246	0.15	0.08	42.7	19.0	8.6	6.7
	2	Rydberg	402.10	0.0315	0.16	0.08	44.2	19.2		
	3	Rydberg	402.30	0.0168	0.18	0.07	43.5	19.1		
	4	Rydberg	402.78	0.0019	0.11		42.8	19.0		
O	1	*s* → π*	533.01	0.0175	0.15	0.06	42.6	19.4	12.1	6.3
	2	Rydberg	533.95	0.0168	0.15	0.16	44.6	19.7		
	3	Rydberg	534.23	0.0000	0.11		40.6	19.2		
	4	*s* → π*	534.29	0.0034	0.10		41.1	19.2		
average					0.14	0.08	42.4	18.8		6.9

a Note that only *f* > 0.015 values are considered in the statistics regarding the oscillator strengths.

#### 4.3.2 Demonstration of the Efficiency

To test the upper limit of our approach, we calculated the four lowest C K-edge excitations of the TIPS-pentacene molecule using our reduced-cost algorithm. This extended system contains 100 atoms and 3366 atomic orbitals using the aug-cc-pVTZ basis set. Therefore, such demanding CVS-ADC(2) calculations are prohibitive or at least highly challenging with conventional implementations. The results are compiled in Table 4. Looking at the wall-clock times required for the main steps, a couple of interesting observations can be made. First, one of the most demanding steps is the calculation of the canonical three-center integral list. That is, the total NAF construction takes 23 h, of which 19 h are spent on integral calculations. Of course, this step should be carried out only once during the procedure, independently of the number of excited states. Second, the wall-clock times required for the VNO and final NAF construction are comparable to those needed for the CVS-ADC(2) iterations. Finally, the solution of the CVS-CIS equations is almost as demanding as the CVS-ADC(2) problem. To be fair, we would like to emphasize that an out-of-core algorithm was executed for the CVS-CIS iterations as the integrals, even in the compressed NAF basis, do not fit into the main memory. This procedure increases the number of disadvantageous I/O operations. Nonetheless, on the basis of these experiences, we can state that a single core excitation can be computed within a bit more than half a day for molecules of 100 atoms using aug-cc-pVTZ basis sets.

**Table 4:** Reduced-Cost CVS-ADC(2) Excitation Energies (ω, in eV) and Oscillator Strengths (*f*) for TIPS-Pentacene Using the aug-cc-pVTZ Basis Set; As Well As the Percentage of VNOs and NAFs Retained and Total Wall-Clock Times (*t*, in Hours) Required for the Main Steps

state	character	ω	*f*	retained VNOs	retained NAFs	*t*_NAF_^a^	*t*_CIS_^b^	*t*_VNO+NAF_^c^	*t*_ADC(2)_^d^
1	*s* → π*	286.84	0.0016	35.5	15.8	23.1	12.5	2.3	3.6
2	*s* → π*	286.84	0.0685	35.5	15.8			2.2	4.3
3	*s* → π*	286.85	0.0007	35.5	15.8			2.3	5.7
4	*s* → π*	286.85	0.0316	35.5	15.8			2.2	5.5

a Steps 1 and 2.

b Step 3.

c Steps 4.a and 4.b.

d Step 4.c in Table 2

## 5 Conclusions

Our frozen VNO- and NAF-based schemes^59^ have been extended to core excitations. The theoretical background of the approximations was briefly presented, and the required modifications compared to the genuine approaches developed for valence-excited states were discussed. The efficiency of the approximations has been demonstrated for the CVS-ADC(2) method. The working equations and algorithmic considerations have also been presented for the corresponding method invoking the well-established density fitting approach.

The errors introduced by the various approximations have been comprehensively analyzed for a representative test set, including molecules from the recently proposed XABOOM test set.^42^ In contrast to the original benchmark set, our compilation contains 1*s* → π* and Rydberg excitations as well. In total, more than 200 excitation energies and 80 oscillator strengths were included in the statistics. On the basis of the results obtained, it can be stated that the combined VNO + NAF approximation is an ideal candidate for reducing the computational requirements. That is, the mean absolute errors are well-balanced for the C, N, and O K-edge excitations, and they do not exceed 0.20 eV. In addition, significant differences in the 1*s* → π* and Rydberg transitions cannot be observed. Concerning the oscillator strengths, the mean relative errors are between 0.06 and 0.08. These values are somewhat more notable than those obtained for excitation energies; nevertheless, the performance is still highly acceptable.

The improvements in the computation times were measured for extended molecules. Using the default cutoff parameters, on average, more than 60% of the VNOs were neglected, while the NAF basis was compressed by a factor of 5. These reductions result in a 7-fold improvement in the wall-clock times and a 30-fold cut in the memory requirements compared with the conventional implementation. Our calculations also demonstrate that using the new approach, the core-excited states of extended systems with 100 atoms can be routinely studied using reliable basis sets at the CVS-ADC(2) level.

## Supporting Information Available

Computed excitation energies and oscillator strengths (XLSX)
Ground-state molecular geometries for the extended molecules (ZIP)
